# Vacuum Processing Technique for Development of Primary Standard Blackbodies

**DOI:** 10.6028/jres.104.018

**Published:** 1999-06-01

**Authors:** M. Navarro, S. S. Bruce, B. Carol Johnson, A. V. Murthy, R. D. Saunders

**Affiliations:** National Institute of Standards and Technology, Gaithersburg, MD 20899-0001

**Keywords:** blackbody sources, fixed-point blackbodies, ITS-90, radiance temperature, rf furnace

## Abstract

Blackbody sources with nearly unity emittance that are in equilibrium with a pure freezing metal such as gold, silver, or copper are used as primary standard sources in the International Temperature Scale of 1990 (ITS-90). Recently, a facility using radio-frequency induction heating for melting and filling the blackbody crucible with the freeze metal under vacuum conditions was developed at the National Institute of Standards and Technology (NIST). The blackbody development under a vacuum environment eliminated the possibility of contamination of the freeze metal during the process. The induction heating, compared to a resistively heated convection oven, provided faster heating of crucible and resulted in shorter turn-around time of about 7 h to manufacture a blackbody. This paper describes the new facility and its application to the development of fixed-point blackbodies.

## 1. Introduction

Sources that are designed to radiate as ideal blackbodies are of fundamental importance in precision radiation thermometry. A blackbody designed to be in equilibrium with pure substances is termed a “fixed-point” blackbody source. The International Temperature Scale of 1990 (ITS-90) [1] establishes temperatures in terms of the equilibrium phase state of pure substances that define the fixed points in the scale. Above the freezing point of silver (1234.93 K), thermodynamic temperature is realized by optical radiation measurements by comparing the spectral radiance of a blackbody at an arbitrary temperature with reference to silver, gold, or copper fixed-point blackbody sources. Fixed-point blackbody sources are also constructed using other pure metals (e.g., tin, zinc, or aluminum) for use in radiation thermometer calibration over a range of temperatures. For fixed-point blackbody sources, the emittance and temperature are known, so the absolute spectral radiance *L*(λ) is calculable at the wavelength λ of interest. The emittance is known from calculations and the temperature from the values assigned in ITS-90.

In contrast to the ITS-90, NIST uses a substitute radiance temperature scale based on direct determination of the spectral radiance of a gold-point blackbody source using detector standards [2]. Combined with the emittance calculations, the measurement resulted in a radiometric determination of the freezing point of gold [3]. The freezing point (1337.33 K) determined by this approach is in agreement with the ITS-90 assignment. Therefore, the NIST radiance temperature scale and the ITS-90 are consistent. At NIST, gold-point blackbody standards are used to derive spectral irradiance and radiance in addition to radiance temperature.

Theoretically, an ideal blackbody source consists of an enclosed isothermal cavity. The radiation within the cavity is in thermal equilibrium with the cavity walls and is determined by the wall temperature only, and the cavity shape has no effect. However, in practical applications, it is necessary to have an aperture in the cavity wall to permit observation and measurement of the radiant flux. The presence of an aperture creates a departure from ideal conditions, which is a small effect for well designed blackbody cavities. Extensive theoretical and computational tools are now available to design a cavity geometry for which the emittance from the aperture is nearly unity [4]. It is also possible to account for non-isothermal wall conditions, surface properties, and other variations from ideal conditions [5].

Practical realization of blackbody sources that can be used as primary standards in precise and accurate radiation and radiance temperature measurements require careful design and manufacturing considerations. Pure metals in a transition phase surrounding a cavity are used to produce stable radiation from the aperture at a fixed point in the temperature scale depending on the metal used. Commercially available high-purity metals, with metallic impurities less than 1 μg/g, are used as freeze metals in the manufacture of primary standard blackbodies. Usually, the lowering of the melt temperature due to low-level impurities is less than about 0.1 mK [6].

NIST designs and builds primary standard blackbody units for its own use and for other standard laboratories involved in the establishment of radiance temperature scales. The blackbody unit is comprised of a sealed annular graphite crucible surrounding the radiating cavity. The crucible containing the freeze metal is used in a heat-pipe furnace. A critical aspect in the manufacture of a blackbody unit is the crucible filling process with the freeze metal. The process requires transferring the pure metal in shot form from a transfer container to the internal volume of the crucible. The internal volume is nearly full when the metal is liquid, but the amount of metal shot required displaces a volume larger than the allowed space. Therefore, the shot must be melted into the crucible, as if it were a mold. In the past, NIST has performed this operation using the actual heat-pipe furnace that forms the rest of the blackbody unit. Melting the shot into the crucible was done in multiple operations to ensure proper filling of the crucible internal volume.

A number of blackbody units were successfully built using the process described above. The process produced high-quality fixed-point blackbodies as demonstrated by experimental studies. However, it lacked the ability to control contamination of the freeze metal during the filling process, because the process was not carried out under vacuum conditions. To overcome this deficiency, a new facility was exclusively developed with the capability of filling the crucibles under high-vacuum conditions. The new facility uses induction heating of the freeze metal in a high-vacuum environment and permits filling the crucible volume with the freeze metal in a single operation. The technique eliminates the risk of contamination during the filling process and helps retain the high purity of the freeze metal. The completed crucible unit is then installed in the heat-pipe furnace for use as primary standards. This paper describes the new facility and its application to the production of fixed-point blackbodies under vacuum conditions.

## 2. Facility Description

**Fig. 1:**
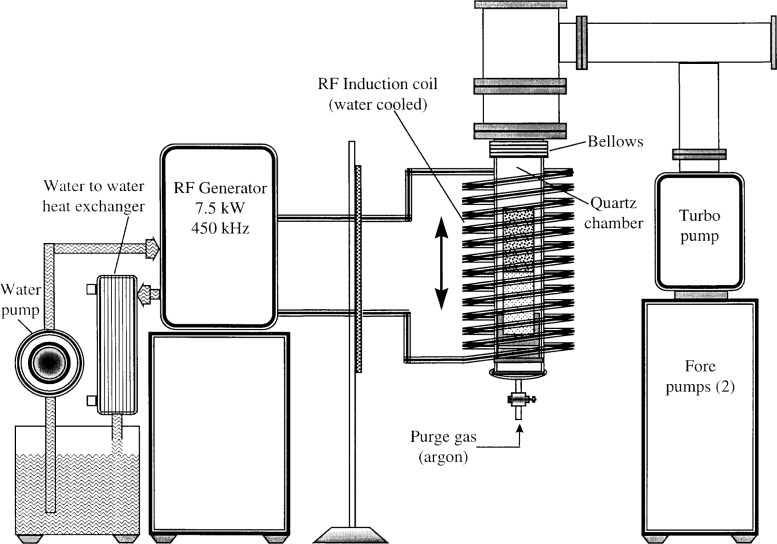
Schematic layout of the vacuum processing facility.

The facility consists of three distinct systems: a radio-frequency (rf) generator with its associated induction coil, a high vacuum system, and a quartz chamber. The quartz chamber houses the crucible and the fill tube containing the freeze metal. Figure 1 shows a schematic layout of the facility.

The rf generator^1^,^2^ used in the induction-heating system is of an industrial type designed for an output power of 7.5 kW at 450 kHz. The unit is housed in a metal cabinet to shield harmful radiation. It operates on a 460 V, 60 Hz, single-phase power supply with a maximum line current of 40 A and an input power of 18 kW. The high-frequency induction current is produced by a vacuum-tube oscillator with a water-cooled triode and has four silicon diodes in the rectifier circuit. The induction coil used for heating the test specimens is made of 1.0 cm outside diameter copper tube. The coil length and mean diameter are 30 cm and 10 cm, respectively, and the coil has 13 turns. The rf unit and the induction coil are water-cooled. The water-cooling system is an integral unit designed primarily to cool the rf unit with all the controls preset. This unit contains a water-to-water heat exchanger, a recirculating pump, and a temperature control valve. The recirculating distilled water is temperature controlled at a preset value of 38 °C.

The air-cooled vacuum system is comprised of a turbo-molecular pump^3^, two oil-sealed rotary fore-pumps, and a vacuum-gauge controller. The nitrogen-equivalent volume flow rate is 200 L/s and the system has sufficient capability for an ultimate pressure of 10^−7^ Pa. To reach this vacuum level, it is necessary to degas the system thoroughly and bake the inlet at 120 °C. The blackbody development process is usually carried out in the pressure range 10^−5^ Pa to 10^−6^ Pa. Pressure measurements are made using a convectron gauge (Granville-Phillips) in the range 10^−2^ Pa to 10 Pa, and an ion gage in the range 10^−7^ Pa to 10^−1^ Pa.

The quartz chamber is a custom made 76 mm diameter, 30 cm long vertical tube. The interior of the tube functions as an oven to heat the graphite crucible and fill tube assembly for fabrication of the blackbody. The entire assembly is installed in the annular space of the rf induction-heating coil described above. The induction coil can be moved vertically with respect to the quartz chamber to provide the best location for heating of the crucible inside the quartz chamber. One end of the quartz tube is fitted with a bellows and a vacuum compatible quick-disconnect stainless steel flange which mates to the vacuum system inlet through stainless steel piping. The other end of the tube, which is concave in shape, is fitted with a stem valve connecting to argon gas supply cylinders. This end also houses the graphite/alumina base support for mounting the crucible/fill tube assembly. Argon gas is used for purging the system and also to bring the quartz chamber pressure from vacuum to ambient conditions to facilitate handling of the assembly during the blackbody-development process.

## 3. Operational Features

The efficiency of the inductive coupling is dependent on the current in the coil, number of turns in the coil, and the shape of the work specimen and its proximity to the coil conductors. In the present set up, only one rf coil with 13 turns is used. The shape of the work specimen, which consists of the crucible and the fill tube, is cylindrical with a diameter of 4.3 cm and a length of 21 cm. The initial phase of commissioning the facility involved determination of appropriate rf current settings to obtain heating levels required for melting different freeze metals. This was done by measuring the work specimen temperature at three locations (top, middle, and bottom) along the specimen length. The temperature was measured using an infrared pyrometer^4^ by sighting the specimen through the quartz chamber wall in the clear view region between the turns of the induction coil.

**Fig. 2:**
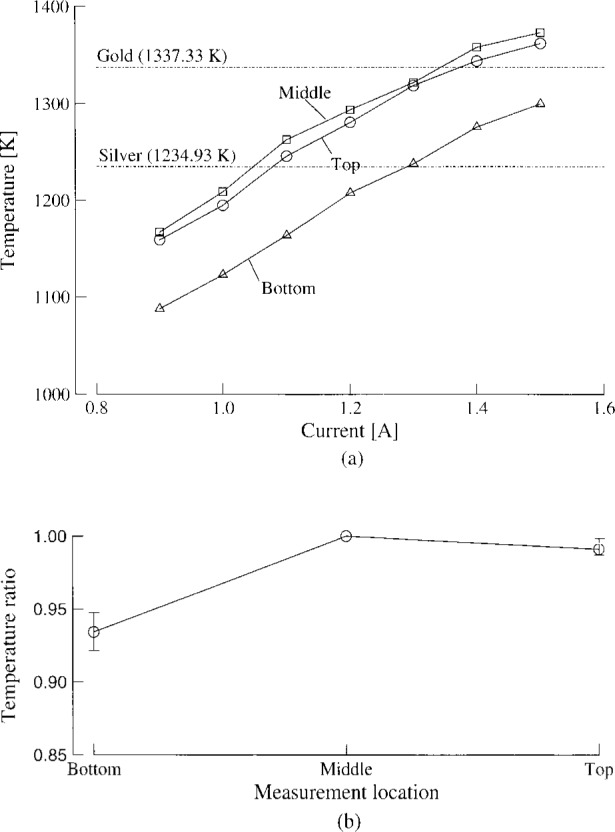
Induction heating facility performance: (a) typical temperature variation at three locations with increasing oscillator plate current; (b) mean temperature distribution referenced to the value at the middle station.

Heating power was varied by increasing the rf generator oscillator plate current. Figure 2a shows a typical measured temperature distribution at three representative locations; top, middle, and bottom, along the length of the work specimen versus increasing plate current. The measurements show that the present coil configuration and the load matching conditions are satisfactory to provide enough heating to melt most of the metals commonly used in high temperature fixed-point blackbodies.^5^ The required heating levels were obtained well within the maximum input plate current in the rf power stage of 2 A.

The temperature distribution appears nearly uniform from the top to the middle section. The bottom section corresponding to the lower portion of the crucible was always cooler relative to middle section. This is probably because the bottom section is supported on a much larger base, resulting in higher heat losses. Figure 2b shows the temporally-averaged temperature distribution over the range of input plate currents between 0.9 A and 1.5 A. The fractional temperature variation is within 1 % at the top of the assembly. The relative drop in temperature at the lower end of the assembly was found to be about 4 % to 8 % of the middle-section temperature. The observed temperature gradients are not critical because the metal completely melts; it is only necessary to ensure that the heating level is adequate to melt the metal. Hence, the performance data in Fig. 2a is used to determine the approximate plate current setting. The actual temperature is monitored continuously using the pyrometer during the blackbody development process.

## 4. Blackbody Development Process

**Fig. 3:**
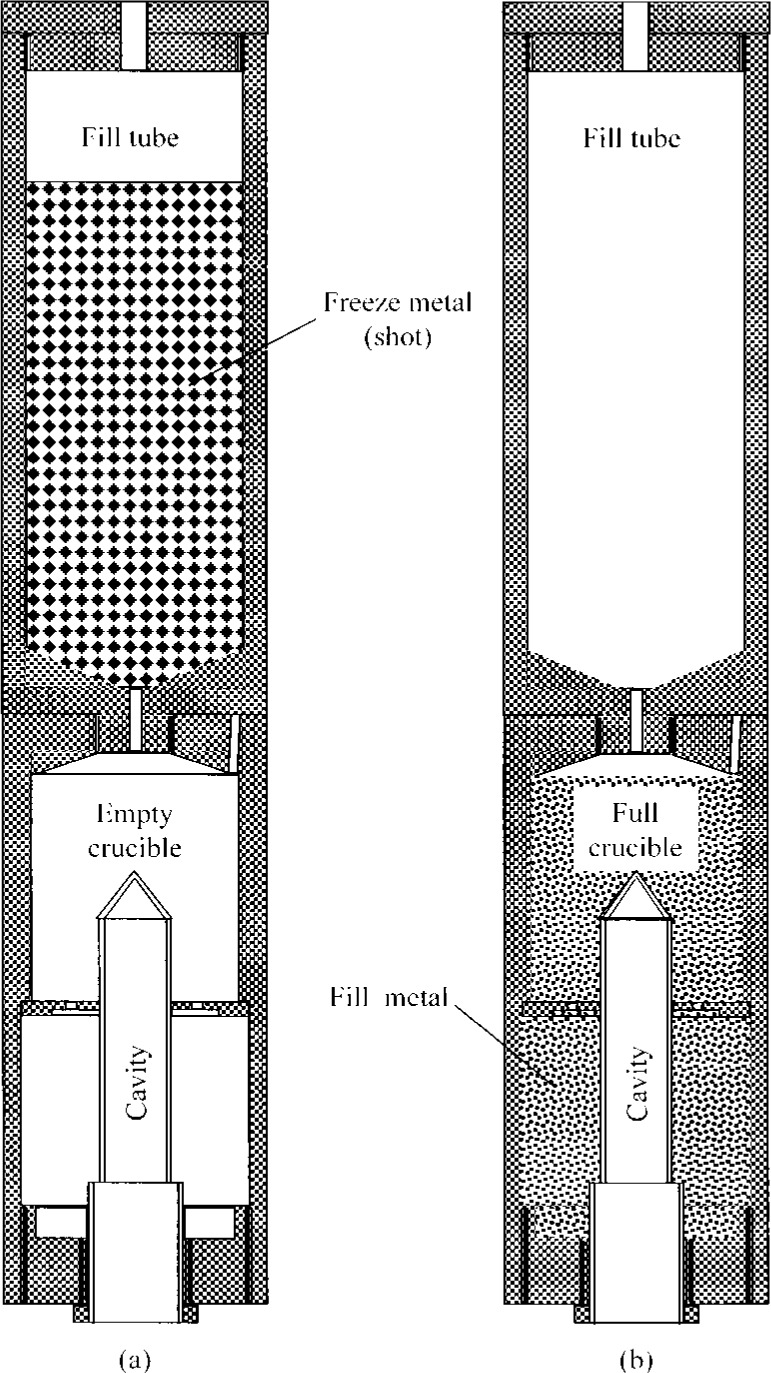
Graphite fill tube and freeze metal crucible assembly: (a) before fill; (b) after fill.

The cavity and crucible parts of the fixed-point blackbodies are constructed from high purity graphite with spectrographic impurities of less than 10^−5^ μg/g. Figure 3 shows a typical assembly of the cavity, the crucible, and the fill tube used in the present process. One end of the blackbody crucible contains the radiating cavity; the other end has a threaded hole. First, the crucible outer shell and cavity parts are assembled to form an empty annular space surrounding the cavity, using threads machined into the parts. Then, the fill tube is attached to form the fill tube/crucible assembly. The fill tube, which is also made of high-purity graphite, is used to contain the freeze metal (in shot form) before melting it and filling the crucible. For the present blackbody cavity configuration, the calculated total fill volume is 65.3 cm^3^, allowing for the increase in volume in the liquid state and adjusted to slightly underfill the crucible volume by about 1 %. This leaves a small a gap above the liquid level and the inside top of the crucible. The freeze metals used are 99.9999 % pure (the mass fraction of all metallic impurities is less than 1 μg/g) and are commercially available in the shot form.

The complete fabrication of the blackbody is done in three steps. In the first step, the quartz chamber containing the empty-crucible/fill-tube assembly without the freeze metal is installed in the facility for decontamination by baking at a high temperature. The induction coil is then positioned around the quartz chamber. After starting the vacuum system, the rf generator is turned on. For gold, the plate current is gradually increased until the graphite temperature reaches approximately 1373 K. The decontamination process is continued for about 2 h or until the pressure reaches 10^−5^ Pa. Then, the induction heating is switched off and the system allowed to cool to ambient temperature. After the system cools, the chamber is isolated from the vacuum pump and the quartz chamber purged with argon gas by opening the valve at the bottom of the chamber. The quartz tube assembly is then removed from the system.

In the second step, the crucible/fill-tube assembly is removed from the quartz chamber. The interior of the quartz chamber is cleaned by soaking in methanol and rinsing with acetone. The measured freeze metal, in shot form, is placed into the tube and the entire assembly placed on the graphite/alumina base inside the quartz chamber. Care is taken in handling the components with suitable disposable gloves to avoid contamination by dirt, grease, or salt from the hands.

In the final step, the quartz chamber is reinstalled in the facility, and the induction coil is positioned around the chamber. After purging the chamber with argon gas, the vacuum system is switched on. When the pressure is below 5.5 × 10^−5^ Pa, the rf generator is turned on and the plate current slowly increased until the measured crucible temperature is above the melting point of the freeze metal. The molten metal starts flowing down by gravity and fills the annular space in the crucible. The temperature is held constant for at least 30 min to ensure complete transfer of the molten metal to the crucible. The chamber temperature is then gradually reduced, and the system is allowed to cool down slowly over a period of 4 h, while under vacuum. After the system has cooled down, the chamber is again isolated from the vacuum pump and the quartz chamber is purged with argon gas to bring it to ambient conditions before removing it from the facility. The total time for steps 1 to 3 for the gold- and silver-point blackbodies developed so far was about 7 h.

**Fig. 4:**
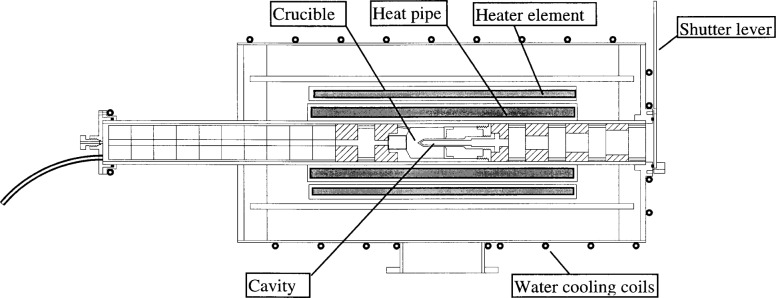
Completed gold-point blackbody unit.

Finally, the fill tube is removed from the assembly and the threaded hole at the crucible back end is fitted with a graphite plug. The finished crucible containing the freeze metal is then installed in a sodium heat-pipe furnace for use as a primary standard in the laboratory. Figure 4 shows a schematic layout of the completed unit as installed in the furnace and ready for delivery. The heat pipe is heated by two semi-cylindrical ceramic heater elements. The unit is operated with argon gas purging to avoid oxidation of graphite cavity and other components. For this cavity configuration, the emissivity of the radiating aperture is calculated to be 0.9999 [3].

## 5. Blackbody Operation

**Fig. 5:**
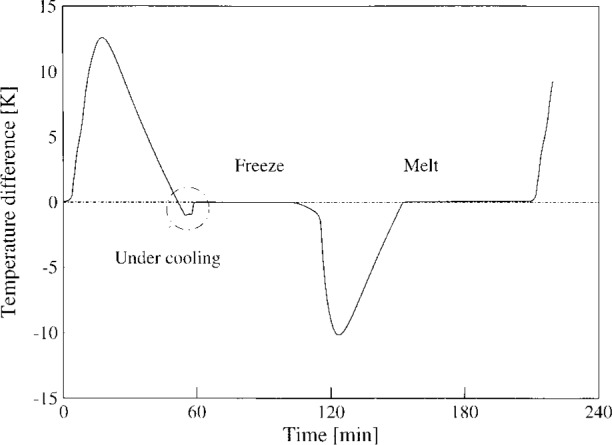
Typical freeze/melt cycle during a measurement of a gold-point blackbody source.

One of the gold-point blackbodies manufactured by the above technique is being used in the NIST Optical Technology Division as a primary standard source [7]. For operation during calibration, the blackbody is first heated gradually to near melt temperature over a period of 8 h at a constant current of 8 A. Then the current is increased to 8.5 A to increase the temperature to about 13 K above the melt temperature, and the freeze metal begins to melt. Following full melting, the freeze cycle (during which the measurements are made) is initiated by lowering the current level to 7.95 A. Successive melting and freezing cycles are obtained by raising and lowering the current between 8.5 A and 7.95 A, respectively. Figure 5 shows an example of a melting and freezing cycle history during a typical calibration. The duration of each melt and freeze interval is about 40 min. Extensive short-term measurements [8] with a germanium radiometer have shown that the freezes repeat and that the drop in temperature during the freeze cycle is about 0.03 K over an interval of 30 min.

## 6. Conclusions

The description of a new radio-frequency induction heating facility for the development of primary standard fixed-point blackbodies under vacuum conditions is presented. The filling of the blackbody crucible with the freeze metal under high vacuum conditions inside a quartz chamber oven greatly reduces the risk of contamination. The new technique presents an improvement over the previous methods using a conventional oven with inert-gas purging. However, to what extent the vacuum process has been beneficial in retaining the purity of the metal is hard to assess without extensive testing of the crucible by different techniques. In the near future, we plan to perform detailed radiometric characterization of the fixed-point blackbodies manufactured so far by the new technique. The resulting data will form a basis for intercomparison with measurements from blackbodies manufactured by other techniques. Such intercomparisons should provide indirect evidence of the improvement and also possibly demonstrate the need for more extensive testing.

## Footnotes

1 Certain commercial equipment, instruments, or materials are identified in this paper to foster understanding. Such identification does not imply recommendation or endorsement by the National Institute of Standards and Technology, nor does it imply that the materials or equipment are necessarily the best available for the purpose.

2 Manufactured by Pillar Industries, Inc., Menomonee Falls, WI.

3 Manufactured by Osaka Vacuum, Ltd., Japan.

4 Minolta Cyclops, Model 52.

5 Copper, gold, silver, aluminum, zinc, and lead.
